# Postpartum Hemorrhage1Presidential address delivered at the annual meeting of the Buffalo Academy of Medicine, June 14, 1904.

**Published:** 1904-09

**Authors:** J. W. Grosvenor

**Affiliations:** 118 Plymouth Ave., Buffalo, N. Y.


### BUFFALO MEDICAL JOURNAL.

VOL. XLIV.—LX. SEPTEMBER, 1904. No. 2

### ORIGINAL COMMUNICATIONS.

### Postpartum Hemorrhage.[1]

By J. W. GROSVENOR, M. D., Buffalo, N. Y.

DEFINITION.

IT IS difficult to formulate an exact definition of postpartum hemorrhage. A definition may comprehend too much or exclude too much to be acceptable to the majority of physicians. A hemorrhage occurring immediately after the termination of labor and sufficiently large to endanger the life of the mother is regarded by all accoucheurs as pathologic and is appropriately termed a postpartum hemorrhage. A small amount of hemorrhage from the placental site, insufficient to sensibly disturb the normal circulation may be properly regarded as physiologic and cannot be appropriately placed among postpartum hemorrhages. Between these two extremes occur hemorrhages of varying quantities about which there may be an honest difference of opinion as to their proper classification. Each medical attendant claims the right to determine whether any particular case of hemorrhage may be termed postpartum.

Cases of hemorrhage which produce a marked disturbance of the circulation and a sense of weakness on the part of the patient are regarded by the writer as cases of postpartum hemorrhage. The term flooding applied to these cases is expressive and readily understood.

The scope of this paper is to deal only with those hemorrhages which proceed from the internal surface of the uterus and especially from the placental site.

1. Presidential address delivered at the annual meeting of the Buffalo Academy of Medicine, June 14, 1904.

### GRAVITY.

The extreme gravity of postpartum hemorrhage is recognised by every obstetrician who has witnessed such a condition. Standing at the bedside where the long expected advent of the newcomer has brought joy to the parents and friends he hears a gurgle as of a rush of water; his hand upon the uterus detects no contraction of that organ; the mother's face is blanched and expressive of anxiety; her pulse is weak and rapidly becomes fluttering; respiration is labored and soon there is gasping for breath; the patient calls for air and says she is cold and dying; windows and doors are thrown open; fans are brought into requisition; ammonia is applied to the nostrils; there is a general excitement among the attendants; as the patient sinks lower and lower a distant sob is heard here and there in the room, the outburst of pent up feelings of relative or friend; the appeals to the medical attendant to save the patient are heart-rending; on investigation he finds the bed almost literally filled with large masses of coagulated blood.

This picture drawn from nature is truly appalling. The contrast between present grief and the joy of a few minutes ago is overwhelming to the stoutest hearts. No wonder that the physician's countenance is white with fear, his throat dry and his heart beats violently with the heavy load of responsibility which he bears. This subject is important, not only to the expert obstetrician, but also to the general practitioner. Any day or any hour any physician may be summoned to an obstetrical case in which postpartum hemorrhage will jeopardise the life of the mother. At such a time energetic promptness, a resourceful mind and a skilful hand are in instant demand.

### CLASSIFICATION.

All kinds of puerperal hemorrhage after the delivery of the child may be divided into two classes—namely, primary and secondary. A hemorrhage which occurs immediately after delivery and is stopped for 24 hours by a contraction of the uterus may be termed appropriately a primary hemorrhage; puerperal hemorrhages which take place after that lapse of time may be regarded as secondary. This classification is arbitrary but perhaps is as useful as any that can be made. Postpartum hemorrhage also may be divided into internal and external, according as the blood remains within the uterine cavity or is expelled from it.

### FREQUENCY.

The frequency of postpartum hemorrhage is not easily determined. This difficulty arises mainly because many births are not reported and deaths from this kind of hemorrhage frequently are reported as due to some other cause. Hence, the unreported births and the wrongly reported deaths are not available as a basis for accuracy in determining the frequency of postpartum hemorrhage. It is generally conceded that this kind of hemorrhage is of rare occurrence in cases of confinement. According to Herman, Guy's Hospital reports present but 1 dangerous case in 2,040 labors and the report of Saint Thomas's Hospital, 1 in 2,172. Hegar reports for Prussia, 1 case in 3,131 labors. It is the opinion of Studer, of Basel, that severe hemorrhage after birth occurs in 5 per cent. of all puerperal cases. Dr. C. S. Bacon, of Chicago, taking the ratio of Studer as a basis and using 60,000 as the annual birth-rate, finds that 3,000 cases of postpartum hemorrhage occur in that city yearly. These figures seem to the writer to be greatly exaggerated.

### DEATH RATE.

According to Bacon, of Chicago, heretofore quoted, the number of puerperal deaths in that city annually is 300. He estimates that of this number, 30 were the result of postpartum hemorrhage. This ratio would give 1 death in every 100 cases of postpartum hemorrhage. Some authors give 1 death in every 300 cases. Dr. Thomas More Madden declares that, after an obstetric practice of twenty years in various countries and in the largest lying-in hospital of Great Britain, he has seen only one death from hemorrhage after childbirth. Even these figures, though not large, emphasize the importance of a more rigid vigilance in prophylaxis and intelligent skill in treatment.

### CAUSES.

These are many and various. A full recital of them will not be attempted in this paper. They may be classified under the following heads: uterine atony; mechanical obstruction; meddlesome midwifery; systemic derangement, and traumatism. Whatever interferes with or prevents a closure of the bleeding vessels may be a cause of postpartum hemorrhage. The interference may be so slight that the bleeding cannot be termed appropriately postpartum hemorrhage. The closure of the ruptured vessels is dependent upon contraction of the uterine musculature. Any systemic condition which wholly or in part prevents this contraction is a factor in the production of postpartum hemorrhage.

*Uterine atony.*—By far the most common cause which prevents uterine contraction is uterine atony. Uterine atony is a result of any one of various causes. There may exist debility of the general system which weakens the uterine muscle; the uterus may lack proper innervation as a result of frequently recurring pregnancies; a tedious labor from any cause may exhaust the uterus to such an extent that it loses the power to contract, as seen in cases which offer any mechanical obstruction to the delivery of the child, and in cases in which the presence of an excessive amount of amniotic fluid produces infrequent and inefficient pains. Local atony may be the effect of paralysis of the placental site.

*Mechanical obstruction.*—A retained placenta or clots obstructing the os uteri; morbid adhesion of placenta; tumors, whether within or without the uterus, as polypi or fibroids; distension of either bladder or rectum; plural births in which there is a long interval between the deliveries,—all these conditions and others are factors in the prevention of effective uterine contraction.

*Meddlesome midwifery.*—After watching many weary hours at the parturient bedside the obstetrician desires as quick a release as possible from his tiresome task. The uterus is in a state of atony from a long service of hard work. It demands a rest; it is in no haste to complete the third stage of labor; it fails to contract with force sufficient to separate the placenta from its site. The impatient accoucheur, anxious to assist nature, pulls and tugs upon the cord, kneads the uterus, mayhap introduces his hand into the uterine cavity and tears the placenta from the uterine wall; he feels a sudden gush of warm fluid; to his dismay he has on his hands a genuine case of postpartum hemorrhage. He realises that he has contravened the course of nature. This is apt to be the experience of the young obstetrician who has seldom sat by the obstetrical bed, rather than of the older obstetrician who has superintended hundreds and possibly thousands of cases.

Nature should be allowed to express the placenta in all cases in which she is equal to the task. Two or three hours may elapse before the contractible powers of the uterus have regained force sufficient to throw off the placenta. For the careful obstetrician this is a time of waiting and watching. Nature rarely needs assistance in this work; if she is unequal to its accomplishment, then is the accoucheur's golden opportunity to lend her a helping hand.

*Systemic derangement.*—Pregnancy invites an extraordinary quantity of blood to the generative organs and this accumulation of blood predisposes to puerperal hemorrhage. While this process is necessary and physiologic it may become detrimental, an instance of a physiologic process leading towards a morbific end.

Any condition that will produce uterine congestion may contribute to uterine hemorrhage, as systemic plethora, over-heating, alcoholics, drastic purgatives. A loss of the normal contractility of the uterine bloodvessels may be a factor in the production of postpartum hemorrhage.

Prolonged and profound anesthesia suspends uterine innervation to such an extent that before the uterus has time to recover its contractile energy, blood is poured in large quantities from the open vessels of the placental site. Many obstetricians do not believe that anesthesia is a factor in increasing hemorrhage after childbirth. To be sure, they modify their opinion by the advice that the anesthesia should be moderate, should not be pushed to the depth demanded by a surgical operation. The writer's experience justifies him in delivering the opinion that if the anesthetic is pushed to the extent of doing effective service, more than a physiologic amount of blood is lost and that if the anesthesia is profound, is surgical or approaches near to the surgical condition, there is imminent danger of genuine postpartum hemorrhage.

A changed condition of the blood is a factor in the production of postpartum hemorrhage. A loss of fibrinogen from the system prevents the formation of fibrin to a physiologic extent; the clots of blood usually organised to close the uterine bloodvessels become deficient in firmness and fail to do their appointed service. This changed condition of the blood is notably observed in cases of albuminuria and malaria.

The hemorrhagic diathesis accounts for some cases of postpartum hemorrhage. The factors concerned in the causation of the hemorrhagic diathesis have not been accurately determined. That such a condition exists is clearly proven by well-attested facts. In many cases in which this condition is present ancestral history strongly points to heredity as a prominent causal factor. If a parturient presents this condition and her ancestors for several generations have suffered in the same way, we must conclude that hereditary influence is largely responsible for its origin and development. It is incontestible that postpartum hemorrhage is an outcome of this diathesis.

*Traumatism.*—Uterine inversion when complete and occurring immediately after delivery is apt to be followed by profuse hemorrhage. However, the hemorrhage will not be severe unless the placenta is detached. Doubtless the most common cause of puerperal uterine hemorrhage is imprudent traction on the cord. It may be the result of a rapid labor when the cord is unusually short or encircles some part of the child's body; in such a case, if the placenta is not very firmly attached to its normal site the strain upon the cord may be sufficient to draw down the fundus and invert the uterus. The systemic shock, caused by the uterine inversion, tends to increase hemorrhage by increasing muscular relaxation and decreasing uterine innervation. The author has had under observation only one case of puerperal uterine inversion. Its probable causes were a relaxed condition of uterine muscular fiber and too energetic traction upon the cord. The hemorrhage was profuse.

Too many are the causes of puerperal uterine rupture to be mentioned in full detail in this paper. It may result from any force which abnormally enlarges the uterine cavity and thus greatly distends and weakens its walls; all organic alterations that degenerate and soften uterine tissue; a previous Cesarean operation; all obstructions that increase the difficulties of labor; instrumental interference for the delivery of the child; excessively strong uterine contractions. The hemorrhage of a ruptured uterus occurs, not only from the surfaces of the rent, but also from the vessels of the placental site. The contractions of a ruptured uterus are not uniform throughout the uterine tissue and are inefficient; hence they do not properly close the mouths of the bleeding vessels and flooding is the consequence.

*Internal hemorrhage.*—Postpartum hemorrhage may be internal as well as external. Internal hemorrhage may be the result of some obstruction to the uterine orifice which prevents the escape of blood, as coagula or a part or whole of the placenta.

### SYMPTOMS.

The prominent symptoms of postpartum hemorrhage are a profuse discharge of blood, generally sudden, sometimes immediately after the birth of the child and again after several hours, usually not until the placenta has been dislodged from its site; relaxation of the uterus which can be detected by placing the hand upon the abdomen; the usual signs of severe hemorrhage, as pallor of the face, intense desire for air, gasping respiration, dimness or loss of vision, a rapid, feeble pulse, dilated pupils, ringing in ears, skin bathed in cold sweat, syncope. When these events occur there can be no doubt that the accoucheur has before him a case of postpartum hemorrhage.

### DIAGNOSIS.

The differential diagnosis of external postpartum hemorrhage presents no special difficulties. The enumeration of symptoms made above furnishes a sufficient and certain warning to the obstetrician that he must grapple with a postpartum hemorrhage. The differential diagnosis of internal hemorrhage is sometimes quite difficult. The enlarged abdomen supposed to be caused by an accumulation of blood in the uterine cavity, may be due to expansion of the intestines. Resonance on percussion will indicate that the resonant sound is due to the presence of gas. A distended bladder may simulate a uterus filled with blood; in such a case evacuation of the bladder will prevent a diagnostic mistake. Syncope immediately or soon after child birth may be due to a very rapid labor; in such a case the bloodvessels which have been compressed by the distended uterus are relieved of their compression and receive the blood rapidly from the head, hence faintness sometimes follows. This condition may be relieved by placing the head lower than the rest of the body. If under any circumstances doubt arises as to the existence of internal hemorrhage, a digital exploration of the uterine cavity will change the doubt into certainty.

### PROGNOSIS.

Flooding after labor is an exceedingly dangerous accident; a few minutes may decide a woman's fate. The more profound the uterine inertia, the more abundant will be the hemorrhage. Internal hemorrhage places the patient in more danger than external, because it is more apt to escape detection. In both external and internal hemorrhages a speedy death is indicated by chills or convulsions, increasing dyspnea, prolonged syncope, sharp and continued pains in the loins, vertigo, loss of vision, dilated pupils. In the very large majority of cases recovery may be confidently expected. This result will be secured by the resourceful and ever-ready obstetrician.

### TREATMENT.

The methods which have been employed in the treatment of postpartum hemorrhage may be classified as follows—namely, pressure, astringents, nerve stimulation, miscellaneous. Some of the methods to be mentioned may be appropriately admitted to more than one of these classes. The object of all kinds of treatment is the contraction and retraction of the uterine muscle for the purpose of closing the wide-open mouths of the uterine bloodvessels.

*Pressure.*—Pressure may be made from without and from within the uterus. The hand placed upon the abdomen grasps the uterine walls and compresses them in order to bring their opposite internal surfaces into close contact, when, speaking generally, they will act as ligatures to the bleeding vessels. An aseptic hand may be passed into the uterus, to remove therefrom any portions of placenta or clots which may remain therein and to make firm pressure upon the placental site. The hand should be retained in this position until it is forced from the uterus by contractions of that organ. The latter procedure may be tiresome to the obstetrician, but it has the advantage of direct pressure at exactly the place of difficulty; it is also advantageous, because the hand is always near and ready and can be used on the spur of the moment. Pressure may also be made by forcing the uterus into an anteflexed position. To accomplish this, place one hand on the abdomen behind the uterus and press it forward; at the same time with two fingers of the other hand in the posterior cul-de-sac press the cervix forward. This maneuvre will bring together the inner surfaces of the uterine walls and compress the bleeding vessels.

Another method of pressure is compression of the abdominal aorta, said to have been first advocated by Rüdiger, a practitioner of Tübingen, in 1797. He introduced his hand into the uterus and compressed the aorta through the uterine wall. In 1825, Ulsamer introduced the method of pressure upon the aorta through the abdominal wall. According to the latter method the obstetrician stands on the right side of the patient and depresses the abdominal wall just above the uterus and a little to the left of the median line; the aortic pulsations are felt and continued pressure with two or three fingers at that point will control the hemorrhage. Notwithstanding the fact that the arterial supply from the ovarian arteries cannot be cut off in this way, it has proved to be a very rapid and effective method of controlling uterine hemorrhage; it stimulates the aortic uterine plexus and, even if it affords only temporary relief, time is given for instituting a more permanent method and saves to the patient a large quantity of needed blood. Compression of the aorta is specially indicated in organic disease of the uterus, such as a fibroid tumor. A rubber bag carried into the uterus and filled with either hot or cold water or air will act as an efficient compressor of the uterine bloodvessels. With many obstetricians iodoform gauze packed into the uterine cavity and allowed to remain 24 or 48 hours is a favorite remedy.

*Astringents.*—Salts of iron by injection, swabbing and tamponing have been largely used for the relief of postpartum hemorrhage. In each case before using iron the uterus should be freed from any retained pieces of placenta or clots and hot water injected for thorough washing. Robert Barnes, in 1857, was the originator of its use by injection, or at least was its earnest advocate. At the beginning of this practice he used a combination of ¼ perchloride of iron and ¾ water, but later 1 part iron to 10 or 12 parts water. Iron coagulates the blood in the mouths of the vessels, acts as an astringent to the inner surface of the uterus and promotes uterine contraction. By injection iron is regarded by some obstetricians as a hazardous remedy on account of producing metroperitonitis. The clots formed by the iron may become septic and septicemia has followed. Doubtless the safest method of using iron on the internal surface of the uterus is by a piece of sponge or swab of cotton. Other astringents, as alum, witch hazel, and tannin have been used for the relief of postpartum hemorrhage, but none has had such wide advocacy as some preparation of the ferric salts.

*Nerve stimulation.*—The object of nerve stimulation is to produce contraction of uterine tissue. This is accomplished by means external and internal, as follows: external massage over the uterus; hot water or hot salt solution at a temperature of 110 to 120° F., injected into the uterine cavity through a double current catheter; flapping the abdominal wall with a wet towel; cold water poured upon the abdomen from a height, said to be objectionable because it increases shock, which already exists, but many of the methods used for checking postpartum hemorrhage are promotive of shock; application of ice to the abdomen; introduction of pieces of ice and injection of cold water into vagina and uterus; vinegar by injection into uterine cavity, first advised by Leroux, in 1776, and highly recommended by Penrose in desperate cases; hypodermic injection of ergot, its absorption being too slow when taken by the mouth; the Faradic current, which is quite effective, but generally not available; hypodermic injections of sulphuric ether are said to have been used with remarkable success in some cases of collapse.

*Miscellaneous.*—Transfusion in nearly all cases is impracticable. However, cases are reported in which life was saved after an unsuccessful trial of several other methods. Autoinfusion by bandaging the extremities may accomplish the same object as transfusion; pulmonary embolism has occurred from its use. Injections of salt solution, both intracellular and intravenous are of extreme utility; they may be dangerous from too great a quantity of the infused fluid; the blood may thus lose largely its power of coagulation. Small infusions frequently given are preferable to one excessively large infusion. Helpful adjuncts to all kinds of treatment are lowering the patient's head and raising the foot of the bed.

Antiseptic precautions are demanded in all internal manipulations and procedures. It is all-important to secure not only contraction but retraction. Occasionally, even a severe case when left to nature recovers. Such recoveries, however, are so infrequent that they should not be cited as an argument in favor of a do-nothing policy.

*After-treatment.*—This may be as important as the more active treatment used before the suppression of the hemorrhage. The uterus should be maintained in a state of retraction by the hand or a compress and firm binder. The patient should not move nor make muscular exertion, should be kept free from mental emotion. Stimulants may be used for exhaustion. The patient should be watched for hours to see if hemorrhage returns.

Of the many remedies mentioned in this paper it is appropriate to inquire which are the safest and most effective. Doubtless success has been secured by each of them. Each obstetric practitioner has his favorite remedy. If, in a particular case, he is unable to accomplish his purpose by his own special method he can easily select another or several others. All cases of postpartum hemorrhage which appear to be alike or very similar cannot be controlled by the same means. Perhaps the most popular method at the present time is the intrauterine injection of hot water.

The two favorite remedies of the author are the pouring of cold water from a height upon the abdominal wall and the passing of the closed hand, made as aseptic as practicable, into the uterine cavity and using it as a compress. Cold water in a pitcher is quickly obtainable and rapidly usable. Although it floods the patient and the bed, an outside flooding with water is safer than an inside flooding with blood. The obstetric hand is always ready and can be effectively used at a moment's warning. In the author's experience both of these methods have been employed with gratifying success.

SECONDARY HEMORRHAGE.

The term secondary may be applied appropriately to a profuse bleeding which occurs a few hours, a few days or even a few weeks after the primary hemorrhage has ceased. This is a very uncommon accident. Collins, in the Dublin Hospital, found 40 such cases out of 16,654 cases of labor observed by him.

The causes of secondary postpartum hemorrhage are generally a portion of placenta, a clot or fragment of membrane remaining in the uterus. Contamin attributes the cause in 6 cases out of 56 to a coagulum. Other exceedingly rare cases are caused by a placenta succenturiata, a uterine polypus or fibroid, mental emotion, hardened feces, alteration of the blood and various other occurrences. The presence of foreign bodies in the uterus demands their immediate removal, such treatment to be followed by those measures which are appropriate for the relief of postpartum hemorrhage of a primary character.

PROPHYLAXIS.

As preventive medicine is more important than curative medicine, so the prophylaxis of postpartum hemorrhage demands more earnest attention than its treatment. Is it not true that too slight consideration has been given to the puerperal woman both before and during labor? In many cases of postpartum hemorrhage the flooding would not have occurred if proper prophylactic measures had been taken. This statement emphasises the importance of medical superintendence of the pregnant woman from the incipiency of her pregnancy to its close. The physician should familiarise himself thoroughly with the causes of postpartum hemorrhage and should study every pregnant case brought to his attention through both his practical and theoretical knowledge. The physical system should be maintained in a condition as near the normal as possible. All organs should be watched and their disturbances corrected.

Anemia should have special attention whether it arises from albuminuria or any other cause. Any derangement of the blood which disturbs the proper proportion of its constituents, as a subnormal amount of fibrin which will prevent the usual coagulability, should receive vigilant and corrective care. A course of treatment by iron may be specially useful. Calcium, being an essential constituent of fibrin, should have a thoroughly remedial trial in such cases. If inquiry concerning the patient's own or her ancestral history leads to the suspicion of a hemorrhagic diathesis, prophylactic measures should be carried out with thoroughness. In such cases the use of an anesthetic is of very doubtful propriety and, in most cases, should be forbidden. If, during labor, postpartum hemorrhage is a foreseen probability an early evacuation of the amniotic fluid may be advisable, which is specially applicable in cases of hydramnios; ergot should be given and there should be a slow delivery of the fetus.

The points in this paper to which the writer wishes to call special attention may be summarised as follows:

1. The gravity of the subject.
2. Uterine atony the most frequent cause of postpartum hemorrhage.
3. Meddlesome midwifery a prolific cause of postpartum hemorrhage.
4. Profound anesthesia a causal factor of postpartum hemorrhage.

5. The need of watchfulness and alertness on the part of the accoucheur.

6. The treatment demands that the accoucheur be prompt and resourceful.

7. The necessity of not only contraction, but of retraction of the uterus.

8. Although cases left to nature have recovered a do-nothing policy is strongly condemned.

9. The usefulness of prophylactic treatment, especially in cases which indicate the existence of a hemorrhagic diathesis.

10. Heredity a causal factor of the hemorrhagic diathesis.

11. The advantages of medical superintendence of pregnancy from its incipiency to its close.

12. The importance of after-treatment.

---

*Fellows of the Buffalo Academy of Medicine:* In terminating my duties as your presiding officer permit me to tender to you my grateful acknowledgments for your confidence expressed by electing me, one year ago, to the official position which I am about to surrender to my successor. I did not aspire to the position and did not suspect that your esteem would select me for your official leader. The place was accepted with much reluctance. Having accepted it, I resolved to make at least a creditable record. My high resolution was entirely dissipated by a physical disability to which I was subjected a few months following my election and which continues to hold me with a tyrannical grip. Kindly accept my sincere apology that my labors in behalf of the academy have been comparatively fruitless of expected results.

A review of the year's work shows that 19 members have been added to our numbers; 13 members have been dropped from the membership roll; hence the year is closed with a membership of 206. Although numerically our organisation maintains a fairly flourishing condition it has not yet received into its fold more than a moiety of the host of physicians resident in our rapidly growing city. The soundness of our treasury presents a solid reason for hearty congratulations.

The scientific and literary productions presented to the academy during the year may not have met the large expectations of some of our members, but many of them have shown careful observation, extensive thought and excellent literary talent. The pathological section deserves special mention on account of its departure from its usual method of conducting its meetings which have been held in different laboratories where scientific truth has been demonstrated by microscopic slides, statistical charts and bacteriologic specimens.

In relation to the general attendance upon the meetings of the sections and the interest taken in the subjects under consideration, it is evident that the papers read should be the result of careful study and thorough investigation and should be presented in an attractive form. Our efforts should be directed towards attracting rather than compelling interest in all the literary and scientific activities of the academy. It is not exaggeration to assert that from the members of this academy often emanate discussions as thoroughly profitable and papers as profoundly scientific as are heard at the meetings of state and national societies.

It has been my fortune to hear various criticisms of the conduct and status of our academy not only during the official year now closing, but also in years past. Some one criticises us for spending too much time in discussing questions that are not strictly scientific, and too much money in forwarding movements that should be directed and maintained by other medical organisations. It is my opinion that too great vigilance cannot be exercised along this line. The academy was organised for the promotion of medical science and art. Questions relating to the investigation of the conduct of public and private institutions and to the enactment and enforcement of laws, should be relegated to the action of the county society which has a broader field and is more polemic in its character.

The criticism is made that the sections do not elect to official position their most competent members. I have heard this criticism from those members who seldom are seen at the meetings of the sections and apparently take but little interest in their success. No doubt the sections are the bulwark, the stronghold of the academy's work and should secure the energetic support of all our members and the leadership of our foremost ability.

Occasionally a member is heard to say, "The academy is of no account to me. I derive no advantage from it." Generally such members attend only one or two meetings each year and do not place themselves in a position to derive advantages which legitimately belong to their membership. Those academicians who are regularly present at the meetings testify that each year brings to them large profit in social relations, official emoluments and scientific attainments.

Not infrequently the opinion has been expressed that the academy holds too many meetings. It may be that the mental, like the physical appetite, becomes clogged by a too frequent administration of literary and scientific pabulum. It is suggested that this subject be taken into full consideration by the incoming council. Other criticisms of a minor or major magnitude have been heard, but the conditions to which they refer can be removed by intelligent planning and unanimity of action. It is evident that the academy has not reached its highest privileges and possibilities. It lacks that personal interest, that *esprit de corps* which kindles enthusiasm and develops self-sacrifice.

In forecasting the academy's future it should be remembered that its success is dependent upon the medical profession of this city. Our physicians can make it or mar it. A strong and common interest in its welfare will lift it to a much higher plane than it occupies at present. A carping, hypercritical spirit united to persistently divided action will reduce it to a level on which none of us will prefer to stand. Every well-regulated physician in Buffalo should regard the academy as his medical home and should exercise towards it the anxious thought and tender regard closely akin to the feeling which he indulges for his domestic fireside. He should look to it as a spring from which flows social happiness, as a light which may illuminate many dark problems demanding solution in his daily round of professional duty, as an inspiring mentor that will point him to lofty ideals and allure him into paths leading to their attainment.

A pressing need of this institution is a permanent home, a dwelling place that we can call our own. It is a fortunate circumstance that already a permanent fund has been set apart for supplying this positively felt want. Although this fund is small it is steadily growing and is, no doubt, a nucleus to which will be added larger or smaller sums till ere long it will become a fund sufficiently large for the construction of a solid structure, commodious enough to concentrate within its walls all the medical society interests of our city. For the near consummation of this project it is unfortunate that Buffalo's medical fraternity contains so few medical men and women of large wealth. Indeed, it is probable that not a single physician of our city is a millionaire. However, it is possible that many of us by persistent attention and wise diplomacy may open the purse-strings of some large-hearted millionaire who belongs to some other profession or calling.

Buffalo is the western metropolis of the Empire State. Many of its physicians are in the front rank of the medical profession of this country,—indeed, of the world. Our academy should enjoy the like reputation among medical societies. It should be recognised as holding a large place by its scientific productions and its continual progress towards high attainments along all medical lines. It should be the unwavering and untiring effort of our membership to win the affections and coöperation of that portion of our city's medical profession now outside of our ranks. We should constantly keep in view the privilege and duty which are ours to maintain a medical society which will supply the needs of a city which is destined to become one of the very largest in the western hemisphere.

If we are true to the possibilities of the Buffalo Academy of Medicine we may look into its future with the confident expectation that it will be one of the towering medical societies of our country. To this end, fellow academicians, I invoke your broadest intelligence and strongest efforts.

118 Plymouth Ave.

